# Supplementary material for: GNTD: reconstructing spatial transcriptomes with graph-guided neural tensor decomposition informed by spatial and functional relations
Source: Nat Commun. 2023 Dec 13;14:8276. doi: 10.1038/s41467-023-44017-0 (PMC10719260; doi:10.1038/s41467-023-44017-0)
Supplement: Supplementary file 1 — Supplementary information [file 41467_2023_44017_MOESM1_ESM.pdf]

Supplementary Information

1 Optimization of GNTD

The loss of GNTD $\mathcal{L}$ is defined as:

$$\mathcal{L} = \mathcal{L}_{\text{recon}} + \lambda \mathcal{L}_{\text{reg}}, \tag{1}$$

where $\mathcal{L}_{\text{recon}}$ denotes the reconstruction loss, $\mathcal{L}_{\text{reg}}$ denotes the Cartesian product graph Laplacian regularization, and λ is the weight on graph Laplacian regularization term. $\mathcal{L}$ can be optimized by training a 3-layer neural network consisting of embedding layer, nonlinear mapping layer, and nonlinear aggregation layer. Without loss of generality, we only show the derivations ignoring the activation functions.

The reconstruction loss $\mathcal{L}_{\text{recon}}$ is written as:

$$\mathcal{L}_{\text{recon}} = \frac{1}{2} \left\| \mathcal{M} \circledast \left(\mathcal{T} - f_{\text{NTD}} \left(\mathcal{T}; \mathbf{W} \right) \right) \right\|_F^2, \tag{2}$$

where $\mathcal{T}$ is the tensor of raw spatial transcriptomics data, and $\mathcal{M}$ is the mask tensor indicating nonzero entries in the $\mathcal{T}$. f_{NTD} denotes the function approximated by the neural network of GNTD. $\mathbf{W} = \bigcup_m \left\{ \mathbf{W}_m^{(\text{emb})} \right\} \bigcup_m \left\{ \mathbf{W}_m^{(\text{nlin})} \right\} \bigcup \left\{ \mathbf{w} \right\}, \forall m = g, y, x$ is the set of all learnable parameters in the neural network, where $\mathbf{W}_m^{(\text{emb})}$, $\mathbf{W}_m^{(\text{nlin})}$, and $\mathbf{w}$ are the parameters in the embedding layer, nonlinear mapping layer, nonlinear aggregation layer, respectively. $\mathcal{L}_{\text{recon}}$ can be further rewritten as:

$$\begin{aligned}
\mathcal{L}_{\text{recon}} &= \frac{1}{2} \left\| \mathcal{M}_{(g)} \circledast \left(\mathcal{T}_{(g)} - \tilde{\mathbf{A}}_g (\mathbf{w} \odot \tilde{\mathbf{A}}_x \odot \tilde{\mathbf{A}}_y)^T \right) \right\|_F^2 \\
&= \frac{1}{2} \left\| \mathcal{M}_{(y)} \circledast \left(\mathcal{T}_{(y)} - \tilde{\mathbf{A}}_g (\mathbf{w} \odot \tilde{\mathbf{A}}_x \odot \tilde{\mathbf{A}}_g)^T \right) \right\|_F^2 \\
&= \frac{1}{2} \left\| \mathcal{M}_{(x)} \circledast \left(\mathcal{T}_{(x)} - \tilde{\mathbf{A}}_y (\mathbf{w} \odot \tilde{\mathbf{A}}_x \odot \tilde{\mathbf{A}}_g)^T \right) \right\|_F^2 \\
&= \frac{1}{2} \left\| \text{vec}(\mathcal{M}) \circledast \left(\text{vec}(\mathcal{T}) - \mathbf{w} (\tilde{\mathbf{A}}_x \odot \tilde{\mathbf{A}}_y \odot \tilde{\mathbf{A}}_g)^T \right) \right\|_F^2,
\end{aligned} \tag{3}$$

where $\mathcal{T}_{(m)}$ and $\mathcal{M}_{(m)}$ denote the mode-m matricization of tensor $\mathcal{T}$ and $\mathcal{M}$, respectively, and $\text{vec}(\cdot)$ denotes the function reshaping the tensor into a vector.

The derivative of $\mathcal{L}_{\text{recon}}$ with respect to $\mathbf{W}$ in the backpropagation can be computed as:

$$\begin{aligned}
\frac{\partial \mathcal{L}_{\text{recon}}}{\partial \mathbf{W}_g^{(\text{emb})}} &= (\mathcal{M}_{(g)} \circledast (\mathcal{T}_{(g)} - \hat{\mathcal{T}}_{(g)}))(\mathbf{w} \odot \tilde{\mathbf{A}}_x \odot \tilde{\mathbf{A}}_y)\tilde{\mathbf{W}}_g^T \\
\frac{\partial \mathcal{L}_{\text{recon}}}{\partial \mathbf{W}_y^{(\text{emb})}} &= (\mathcal{M}_{(y)} \circledast (\mathcal{T}_{(y)} - \hat{\mathcal{T}}_{(y)}))(\mathbf{w} \odot \tilde{\mathbf{A}}_x \odot \tilde{\mathbf{A}}_g)\tilde{\mathbf{W}}_y^T \\
\frac{\partial \mathcal{L}_{\text{recon}}}{\partial \mathbf{W}_x^{(\text{emb})}} &= (\mathcal{M}_{(x)} \circledast (\mathcal{T}_{(x)} - \hat{\mathcal{T}}_{(x)}))(\mathbf{w} \odot \tilde{\mathbf{A}}_y \odot \tilde{\mathbf{A}}_g)\tilde{\mathbf{W}}_x^T
\end{aligned} \tag{4}$$

$$\begin{aligned}
\frac{\partial \mathcal{L}_{\text{recon}}}{\partial \mathbf{W}_g^{(\text{nlin})}} &= \mathbf{A}_g^T(\mathcal{M}_{(g)} \circledast (\mathcal{T}_{(g)} - \hat{\mathcal{T}}_{(g)}))(\mathbf{w} \odot \tilde{\mathbf{A}}_x \odot \tilde{\mathbf{A}}_y) \\
\frac{\partial \mathcal{L}_{\text{recon}}}{\partial \mathbf{W}_y^{(\text{nlin})}} &= \mathbf{A}_y^T(\mathcal{M}_{(y)} \circledast (\mathcal{T}_{(y)} - \hat{\mathcal{T}}_{(y)}))(\mathbf{w} \odot \tilde{\mathbf{A}}_x \odot \tilde{\mathbf{A}}_g) \\
\frac{\partial \mathcal{L}_{\text{recon}}}{\partial \mathbf{W}_x^{(\text{nlin})}} &= \mathbf{A}_x^T(\mathcal{M}_{(x)} \circledast (\mathcal{T}_{(x)} - \hat{\mathcal{T}}_{(x)}))(\mathbf{w} \odot \tilde{\mathbf{A}}_y \odot \tilde{\mathbf{A}}_g)
\end{aligned} \tag{5}$$

$$\frac{\partial \mathcal{L}_{\text{recon}}}{\partial \mathbf{w}} = (\text{vec}(\mathcal{M}) \circledast (\text{vec}(\mathcal{T}) - \text{vec}(\hat{\mathcal{T}})))(\tilde{\mathbf{A}}_x \odot \tilde{\mathbf{A}}_y \odot \tilde{\mathbf{A}}_g), \tag{6}$$

where $\hat{\mathcal{T}} = f_{\text{NTD}}(\mathcal{T}; \mathbf{W})$ is the tensor of imputed spatial transcriptomics data, and $\hat{\mathcal{T}}_{(m)}$ denotes its mode-m matricization. $\mathbf{W}_m = \mathbf{W}_m^{(\text{emb})}, \tilde{\mathbf{W}}_m = \mathbf{W}_m^{(\text{nlin})}, \forall m = g, y, x$ are used for simplicity of the notations. $\mathbf{A}_m$ and $\tilde{\mathbf{A}}_g, \forall m = g, y, x$ represent the linear and nonlinear factor matrices along m-th mode, respectively.

The Cartesian product graph regularization $\mathcal{L}_{\text{reg}}$ is written as:

$$\mathcal{L}_{\text{reg}} = \frac{\lambda}{2}\text{vec}(f'_{\text{NTD}}(\mathcal{T}; \mathbf{W}))^T \mathbf{L}_c \text{vec}(f'_{\text{NTD}}(\mathcal{T}; \mathbf{W})), \tag{7}$$

where $\mathbf{L}_c = \mathbf{L}_{xy} \oplus \mathbf{L}_g$ denotes Cartesian product graph Laplacian, $\mathbf{L}_{xy}$ and $\mathbf{L}_g$ represents the graph Laplacian of the spatial and PPI graphs, respectively. Note that f'_{NTD} is an estimation to f_{NTD}, which does not involve the activation function in the last layer. $\mathcal{L}_{\text{reg}}$ can be further simplified as:

$$\begin{aligned}
\mathcal{L}_{\text{reg}} = &\frac{\lambda}{2}\mathbf{1}_{\tilde{r}}^T(\mathbf{w}\mathbf{w}^T \circledast \tilde{\mathbf{A}}_g^T\tilde{\mathbf{A}}_g \circledast \tilde{\mathbf{A}}_x^T\tilde{\mathbf{A}}_x \circledast \tilde{\mathbf{A}}_y^T\tilde{\mathbf{A}}_y)\mathbf{1}_{\tilde{r}} \\
+&\frac{1}{2}\mathbf{1}_{\tilde{r}}^T(\mathbf{w}\mathbf{w}^T \circledast \tilde{\mathbf{A}}_g^T\tilde{\mathbf{A}}_g \circledast ((\tilde{\mathbf{A}}_x \odot \tilde{\mathbf{A}}_y)^T\mathbf{L}_{xy}(\tilde{\mathbf{A}}_x \odot \tilde{\mathbf{A}}_y)))\mathbf{1}_{\tilde{r}} \\
+&\frac{1}{2}\mathbf{1}_{\tilde{r}}^T(\mathbf{w}\mathbf{w}^T \circledast \tilde{\mathbf{A}}_g^T\mathbf{L}_g\tilde{\mathbf{A}}_g \circledast \tilde{\mathbf{A}}_x^T\tilde{\mathbf{A}}_x \circledast \tilde{\mathbf{A}}_y^T\tilde{\mathbf{A}}_y)\mathbf{1}_{\tilde{r}}.
\end{aligned} \tag{8}$$

The derivative of $\mathcal{L}_{\text{reg}}$ with respect to $\mathbf{W}$ in the backpropagation can be computed as:

$$
\begin{aligned}
\frac{\partial \mathcal{L}_{\text{reg}}}{\partial \mathbf{W}_g^{(\text{nlin})}} &= (\mathbf{A}_g^T \tilde{\mathbf{A}}_g + \mathbf{A}_g^T \mathbf{L}_g \tilde{\mathbf{A}}_g)(\mathbf{w}\mathbf{w}^T \circledast \tilde{\mathbf{A}}_x^T \tilde{\mathbf{A}}_x \circledast \tilde{\mathbf{A}}_y^T \tilde{\mathbf{A}}_y) \\
&+ \mathbf{A}_g^T \tilde{\mathbf{A}}_g (\mathbf{w}\mathbf{w}^T \circledast ((\tilde{\mathbf{A}}_x \odot \tilde{\mathbf{A}}_y)^T \mathbf{L}_{xy} (\tilde{\mathbf{A}}_x \odot \tilde{\mathbf{A}}_y))) \\
\frac{\partial \mathcal{L}_{\text{reg}}}{\partial \mathbf{W}_y^{(\text{nlin})}} &= \mathbf{A}_y^T \tilde{\mathbf{A}}_y (\mathbf{w}\mathbf{w}^T \circledast (\tilde{\mathbf{A}}_g^T \tilde{\mathbf{A}}_g + \tilde{\mathbf{A}}_g^T \mathbf{L}_g \tilde{\mathbf{A}}_g) \circledast \tilde{\mathbf{A}}_x^T \tilde{\mathbf{A}}_x) \\
&+ \operatorname{vec}_{r\times\tilde{r}}^{-1}(\tilde{\mathbf{C}}_y^T \operatorname{vec}(\mathbf{L}_{xy} (\tilde{\mathbf{A}}_x \odot \tilde{\mathbf{A}}_y)(\mathbf{w}\mathbf{w}^T \circledast \tilde{\mathbf{A}}_g^T \tilde{\mathbf{A}}_g))) \\
\frac{\partial \mathcal{L}_{\text{reg}}}{\partial \mathbf{W}_x^{(\text{nlin})}} &= \mathbf{A}_x^T \tilde{\mathbf{A}}_x (\mathbf{w}\mathbf{w}^T \circledast (\tilde{\mathbf{A}}_g^T \tilde{\mathbf{A}}_g + \tilde{\mathbf{A}}_g^T \mathbf{L}_g \tilde{\mathbf{A}}_g) \circledast \tilde{\mathbf{A}}_y^T \tilde{\mathbf{A}}_y) \\
&+ \operatorname{vec}_{r\times\tilde{r}}^{-1}(\tilde{\mathbf{C}}_x^T \operatorname{vec}(\mathbf{L}_{xy} (\tilde{\mathbf{A}}_x \odot \tilde{\mathbf{A}}_y)(\mathbf{w}\mathbf{w}^T \circledast \tilde{\mathbf{A}}_g^T \tilde{\mathbf{A}}_g)))
\end{aligned} \tag{9}
$$

$$
\begin{aligned}
\frac{\partial \mathcal{L}_{\text{reg}}}{\partial \mathbf{W}_g^{(\text{emb})}} &= (\tilde{\mathbf{A}}_g + \mathbf{L}_g \tilde{\mathbf{A}}_g)(\mathbf{w}\mathbf{w}^T \circledast \tilde{\mathbf{A}}_x^T \tilde{\mathbf{A}}_x \circledast \tilde{\mathbf{A}}_y^T \tilde{\mathbf{A}}_y) \tilde{\mathbf{W}}_g^T \\
&+ \tilde{\mathbf{A}}_g (\mathbf{w}\mathbf{w}^T \circledast ((\tilde{\mathbf{A}}_x \odot \tilde{\mathbf{A}}_y)^T \mathbf{L}_{xy} (\tilde{\mathbf{A}}_x \odot \tilde{\mathbf{A}}_y))) \tilde{\mathbf{W}}_g^T \\
\frac{\partial \mathcal{L}_{\text{reg}}}{\partial \mathbf{W}_y^{(\text{emb})}} &= \tilde{\mathbf{A}}_y (\mathbf{w}\mathbf{w}^T \circledast (\tilde{\mathbf{A}}_g^T \tilde{\mathbf{A}}_g + \tilde{\mathbf{A}}_g^T \mathbf{L}_g \tilde{\mathbf{A}}_g) \circledast \tilde{\mathbf{A}}_x^T \tilde{\mathbf{A}}_x) \tilde{\mathbf{W}}_y^T \\
&+ \operatorname{vec}_{y\times r}^{-1}(\mathbf{C}_y^T \operatorname{vec}(\mathbf{L}_{xy} (\tilde{\mathbf{A}}_x \odot \tilde{\mathbf{A}}_y)(\mathbf{w}\mathbf{w}^T \circledast \tilde{\mathbf{A}}_g^T \tilde{\mathbf{A}}_g))) \\
\frac{\partial \mathcal{L}_{\text{reg}}}{\partial \mathbf{W}_x^{(\text{emb})}} &= \tilde{\mathbf{A}}_x (\mathbf{w}\mathbf{w}^T \circledast (\tilde{\mathbf{A}}_g^T \tilde{\mathbf{A}}_g + \tilde{\mathbf{A}}_g^T \mathbf{L}_g \tilde{\mathbf{A}}_g) \circledast \tilde{\mathbf{A}}_y^T \tilde{\mathbf{A}}_y) \tilde{\mathbf{W}}_x^T \\
&+ \operatorname{vec}_{x\times r}^{-1}(\mathbf{C}_x^T \operatorname{vec}(\mathbf{L}_{xy} (\tilde{\mathbf{A}}_x \odot \tilde{\mathbf{A}}_y)(\mathbf{w}\mathbf{w}^T \circledast \tilde{\mathbf{A}}_g^T \tilde{\mathbf{A}}_g)))
\end{aligned} \tag{10}
$$

$$
\begin{aligned}
\frac{\partial \mathcal{L}_{\text{reg}}}{\partial \mathbf{w}} &= ((\tilde{\mathbf{A}}_g^T \tilde{\mathbf{A}}_g + \tilde{\mathbf{A}}_g^T \mathbf{L}_g \tilde{\mathbf{A}}_g) \circledast \tilde{\mathbf{A}}_x^T \tilde{\mathbf{A}}_x \circledast \tilde{\mathbf{A}}_y^T \tilde{\mathbf{A}}_y)\mathbf{w} \\
&+ (\tilde{\mathbf{A}}_g^T \tilde{\mathbf{A}}_g \circledast ((\tilde{\mathbf{A}}_x \odot \tilde{\mathbf{A}}_y)^T \mathbf{L}_{xy} (\tilde{\mathbf{A}}_x \odot \tilde{\mathbf{A}}_y)))\mathbf{w},
\end{aligned} \tag{11}
$$

where $\mathbf{C}_x = (\mathbf{I}_{x\tilde{r}} \odot (\tilde{\mathbf{A}}_y(\mathbf{I}_{\tilde{r}} \otimes \mathbf{1}_x^T)))(\tilde{\mathbf{W}}_x^T \otimes \mathbf{I}_x)$ and $\tilde{\mathbf{C}}_x = (\mathbf{I}_{x\tilde{r}} \odot (\tilde{\mathbf{A}}_y(\mathbf{I}_{\tilde{r}} \otimes \mathbf{1}_x^T)))(\mathbf{I}_{\tilde{r}} \otimes \mathbf{A}_x)$, and $\mathbf{C}_y = ((\mathbf{I}_{\tilde{r}} \odot \tilde{\mathbf{A}}_x) \otimes \mathbf{I}_y)(\tilde{\mathbf{W}}_y^T \otimes \mathbf{I}_y)$ and $\tilde{\mathbf{C}}_y = ((\mathbf{I}_{\tilde{r}} \odot \tilde{\mathbf{A}}_x) \otimes \mathbf{I}_y)(\mathbf{I}_{\tilde{r}} \otimes \mathbf{A}_y)$. $\operatorname{vec}_{p\times q}^{-1}(\cdot)$ denotes the function reshaping the vector into a matrix of size $n_p \times n_q$.

If we construct the spatial neighbor graph $\mathbf{G}_{xy}$ with all available spots in the array no matter if these spots are overlapped with tissue, its graph Laplacian can be further decomposed into $\mathbf{L}_{xy} = \mathbf{L}_x \oplus \mathbf{L}_y$, where $\mathbf{L}_x \in \mathbb{R}^{n_x \times n_x}$ and $\mathbf{L}_y \in \mathbb{R}^{n_y \times n_y}$ denote the graph Laplacian of chain graphs $\mathbf{G}_x$ and $\mathbf{G}_y$ respectively. And the derivative of $\mathcal{L}_{\text{reg}}$ with respect to $\mathbf{W}$ can be rewritten as:

$$
\begin{aligned}
\frac{\partial \mathcal{L}_{\text{reg}}}{\partial \mathbf{W}_g^{(\text{nlin})}} &= (\mathbf{A}_g^T \tilde{\mathbf{A}}_g + \mathbf{A}_g^T \mathbf{L}_g \tilde{\mathbf{A}}_g)(\mathbf{w}\mathbf{w}^T \circledast \tilde{\mathbf{A}}_x^T \tilde{\mathbf{A}}_x \circledast \tilde{\mathbf{A}}_y^T \tilde{\mathbf{A}}_y) \\
&+ \mathbf{A}_g^T \tilde{\mathbf{A}}_g(\mathbf{w}\mathbf{w}^T \circledast \tilde{\mathbf{A}}_x^T \mathbf{L}_x \tilde{\mathbf{A}}_x \circledast \tilde{\mathbf{A}}_y^T \tilde{\mathbf{A}}_y) \\
&+ \mathbf{A}_g^T \tilde{\mathbf{A}}_g(\mathbf{w}\mathbf{w}^T \circledast \tilde{\mathbf{A}}_x^T \tilde{\mathbf{A}}_x \circledast \tilde{\mathbf{A}}_y^T \mathbf{L}_y \tilde{\mathbf{A}}_y) \\
\frac{\partial \mathcal{L}_{\text{reg}}}{\partial \mathbf{W}_y^{(\text{nlin})}} &= \mathbf{A}_y^T \tilde{\mathbf{A}}_y(\mathbf{w}\mathbf{w}^T \circledast (\tilde{\mathbf{A}}_g^T \tilde{\mathbf{A}}_g + \tilde{\mathbf{A}}_g^T \mathbf{L}_g \tilde{\mathbf{A}}_g) \circledast \tilde{\mathbf{A}}_x^T \tilde{\mathbf{A}}_x) \\
&+ \mathbf{A}_y^T \tilde{\mathbf{A}}_y(\mathbf{w}\mathbf{w}^T \circledast \tilde{\mathbf{A}}_g^T \tilde{\mathbf{A}}_g \circledast \tilde{\mathbf{A}}_x^T \mathbf{L}_x \tilde{\mathbf{A}}_x) \\
&+ \mathbf{A}_y^T \mathbf{L}_y \tilde{\mathbf{A}}_y(\mathbf{w}\mathbf{w}^T \circledast \tilde{\mathbf{A}}_g^T \tilde{\mathbf{A}}_g \circledast \tilde{\mathbf{A}}_x^T \tilde{\mathbf{A}}_x) \\
\frac{\partial \mathcal{L}_{\text{reg}}}{\partial \mathbf{W}_x^{(\text{nlin})}} &= \mathbf{A}_x^T \tilde{\mathbf{A}}_x(\mathbf{w}\mathbf{w}^T \circledast (\tilde{\mathbf{A}}_g^T \tilde{\mathbf{A}}_g + \tilde{\mathbf{A}}_g^T \mathbf{L}_g \tilde{\mathbf{A}}_g) \circledast \tilde{\mathbf{A}}_y^T \tilde{\mathbf{A}}_y) \\
&+ \mathbf{A}_x^T \tilde{\mathbf{A}}_x(\mathbf{w}\mathbf{w}^T \circledast \tilde{\mathbf{A}}_g^T \tilde{\mathbf{A}}_g \circledast \tilde{\mathbf{A}}_y^T \mathbf{L}_y \tilde{\mathbf{A}}_y) \\
&+ \mathbf{A}_x^T \mathbf{L}_x \tilde{\mathbf{A}}_x(\mathbf{w}\mathbf{w}^T \circledast \tilde{\mathbf{A}}_g^T \tilde{\mathbf{A}}_g \circledast \tilde{\mathbf{A}}_y^T \tilde{\mathbf{A}}_y)
\end{aligned} \tag{12}
$$

$$
\begin{aligned}
\frac{\partial \mathcal{L}_{\text{reg}}}{\partial \mathbf{W}_g^{(\text{emb})}} &= (\tilde{\mathbf{A}}_g + \mathbf{L}_g \tilde{\mathbf{A}}_g)(\mathbf{w}\mathbf{w}^T \circledast \tilde{\mathbf{A}}_x^T \tilde{\mathbf{A}}_x \circledast \tilde{\mathbf{A}}_y^T \tilde{\mathbf{A}}_y)\tilde{\mathbf{W}}_g^T \\
&+ \tilde{\mathbf{A}}_g(\mathbf{w}\mathbf{w}^T \circledast \tilde{\mathbf{A}}_x^T \mathbf{L}_x \tilde{\mathbf{A}}_x \circledast \tilde{\mathbf{A}}_y^T \tilde{\mathbf{A}}_y)\tilde{\mathbf{W}}_g^T \\
&+ \tilde{\mathbf{A}}_g(\mathbf{w}\mathbf{w}^T \circledast \tilde{\mathbf{A}}_x^T \tilde{\mathbf{A}}_x \circledast \tilde{\mathbf{A}}_y^T \mathbf{L}_y \tilde{\mathbf{A}}_y)\tilde{\mathbf{W}}_g^T \\
\frac{\partial \mathcal{L}_{\text{reg}}}{\partial \mathbf{W}_y^{(\text{emb})}} &= \tilde{\mathbf{A}}_y(\mathbf{w}\mathbf{w}^T \circledast (\tilde{\mathbf{A}}_g^T \tilde{\mathbf{A}}_g + \tilde{\mathbf{A}}_g^T \mathbf{L}_g \tilde{\mathbf{A}}_g) \circledast \tilde{\mathbf{A}}_x^T \tilde{\mathbf{A}}_x)\tilde{\mathbf{W}}_y^T \\
&+ \tilde{\mathbf{A}}_y(\mathbf{w}\mathbf{w}^T \circledast \tilde{\mathbf{A}}_g^T \tilde{\mathbf{A}}_g \circledast \tilde{\mathbf{A}}_x^T \mathbf{L}_x \tilde{\mathbf{A}}_x)\tilde{\mathbf{W}}_y^T \\
&+ \mathbf{L}_y \tilde{\mathbf{A}}_y(\mathbf{w}\mathbf{w}^T \circledast \tilde{\mathbf{A}}_g^T \tilde{\mathbf{A}}_g \circledast \tilde{\mathbf{A}}_x^T \tilde{\mathbf{A}}_x)\tilde{\mathbf{W}}_y^T \\
\frac{\partial \mathcal{L}_{\text{reg}}}{\partial \mathbf{W}_x^{(\text{emb})}} &= \tilde{\mathbf{A}}_x(\mathbf{w}\mathbf{w}^T \circledast (\tilde{\mathbf{A}}_g^T \tilde{\mathbf{A}}_g + \tilde{\mathbf{A}}_g^T \mathbf{L}_g \tilde{\mathbf{A}}_g) \circledast \tilde{\mathbf{A}}_y^T \tilde{\mathbf{A}}_y)\tilde{\mathbf{W}}_x^T \\
&+ \tilde{\mathbf{A}}_x(\mathbf{w}\mathbf{w}^T \circledast \tilde{\mathbf{A}}_g^T \tilde{\mathbf{A}}_g \circledast \tilde{\mathbf{A}}_y^T \mathbf{L}_y \tilde{\mathbf{A}}_y)\tilde{\mathbf{W}}_x^T \\
&+ \mathbf{L}_x \tilde{\mathbf{A}}_x(\mathbf{w}\mathbf{w}^T \circledast \tilde{\mathbf{A}}_g^T \tilde{\mathbf{A}}_g \circledast \tilde{\mathbf{A}}_y^T \tilde{\mathbf{A}}_y)\tilde{\mathbf{W}}_x^T
\end{aligned} \tag{13}
$$

$$
\begin{aligned}
\frac{\partial \mathcal{L}_{\text{reg}}}{\partial \mathbf{w}} &= ((\tilde{\mathbf{A}}_g^T \tilde{\mathbf{A}}_g + \tilde{\mathbf{A}}_g^T \mathbf{L}_g \tilde{\mathbf{A}}_g) \circledast \tilde{\mathbf{A}}_x^T \tilde{\mathbf{A}}_x \circledast \tilde{\mathbf{A}}_y^T \tilde{\mathbf{A}}_y)\mathbf{w} \\
&+ (\tilde{\mathbf{A}}_g^T \tilde{\mathbf{A}}_g \circledast \tilde{\mathbf{A}}_x^T \mathbf{L}_x \tilde{\mathbf{A}}_x \circledast \tilde{\mathbf{A}}_y^T \tilde{\mathbf{A}}_y)\mathbf{w} \\
&+ (\tilde{\mathbf{A}}_g^T \tilde{\mathbf{A}}_g \circledast \tilde{\mathbf{A}}_x^T \tilde{\mathbf{A}}_x \circledast \tilde{\mathbf{A}}_y^T \mathbf{L}_y \tilde{\mathbf{A}}_y)\mathbf{w}
\end{aligned} \tag{14}
$$

2 Comparisons to reference-based imputation methods

There are other methods using external data such as staining images or single-cell transcriptomics as references for spatial transcriptomics data and they could be potentially applied for imputation as well. Examples of these methods are convolutional neural network (CNN) ST-Net and generative model XFuse which utilizes H&E staining images shipped with spatial transcriptomics, and deep learning model Tangram which uses single-cell transcriptomics measured on a similar tissue region as reference. Despite it is promising to improve imputation by exploring external information, such as nuclei segmentation or convolutional features in staining images, and expression distribution or cell types in single-cell transcriptomics, we were unable to perform a conclusive comparison between GNTD and the state-of-the-art methods in these categories as they have different focuses in modeling.

2.1 ST-Net

ST-Net only relies on convolutional features extracted from a pre-trained DenseNet and fine-tunes the model to predict the expression of the 250 genes with the highest expression rather than the whole transcriptome. Besides, it is unclear if ST-Net is sufficiently expressive to predict the expression of more than $10k$ genes with the $1,024$ convolutional features from the DenseNet. This is a fundamentally different setting compared with GNTD which models non-zero entries in the spatial gene expressions for the whole transcriptome-wide imputation. It is also not reasonable to apply GNTD to impute fewer genes like ST-Net does because GNTD's performance might be undermined by involving the protein-protein network (PPI) consisting of such a small subset of genes. Lastly, ST-Net also needs to be trained with as many H&E image patches over spots as possible. In the original study, the model was trained for a breast cancer tissue dataset with around $20k$ patches from 68 H&E images plus data augmentation. In such a scale, it is impractical to apply ST-Net to the typical Visium or Stereo-seq datasets that usually only have 1 section or 2 replicates. Moreover, in the attempts to comparing GNTD with ST-Net on the datasets we used in our studies, we found that the H&E image quality for the 12 human brain DLPFC replicates from the spatialLIBD project is substantially worse, which resulted in poor performance for the top gene expression prediction.

2.2 XFuse

XFuse is a deep generative model integrating both spatial transcriptomics data and staining images to infer super-resolved expression maps. We attempted to follow the same imputation of a holdout tissue region on the human small intestine in the experiments from the original work for possible comparison. However, imputation performance in their studies was evaluated on only 100 genes with the highest expression for the spots in the holdout tissue region with Pearson correlation. This evaluation would not be suitable for GNTD since such a small set of genes especially for the

highest expressed genes would not benefit from the connections in the whole PPI network. We also expect other obstacles to compare XFuse with GNTD. XFuse by default imputes a $2k \times 2k$ super-resolution expression map. The big discrepancy to the standard size of the Visium array (78×64) or the size of the Stereo-seq array (around 200×200) after binning, leads to doubt on applying XFuse in such a setting. While it is possible to introduce additional post-processing of super-resolution down to the actual size, XFuse does not explicitly model the reconstruction loss and is unlikely to perform well in imputation by the evaluation metrics designed in our studies. Finally, the main challenge in practice is the expected running time. In our testing, XFuse was not scalable on the Stereo-seq datasets. The documentation of XFuse package also specifies that it will take more than three days to train the model on a standard Visium dataset with around $5k$ spots and $10k$ genes on a GPU card with 12GB GPU memory, which poses significant difficulty in fitting XFuse in cross-validation on the datasets for a reliable comparison.

2.3 Tangram

Tangram aims to learn the mapping function between cells in single-cell transcriptomics and spots in spatial transcriptomics by maximizing the similarity of spatial distributions between mapped single-cell expression and spatial expression on a set of marker genes, where the imputed data is single-cell gene expression after applying the mapping function. Since the primary goal of Tangram is cell-type deconvolution rather than precise imputation, the nature of the imputed data is entirely different from the ST expression data. We attempted to compare GNTD and Tangram by performing the same spot-wise and gene-wise 10-fold cross-validation on the Visium data of the mouse brain cortex region with matched scRNAseq data tested in the original study introducing Tangram. We measured the evaluation metrics designed in our studies for both methods and found out that Tangram performed much worse (spot-wise and gene-wise MAE are 7.0494 and 6.7384, respectively, much greater than the results of GNTD and the other baselines shown in Figure 2), which is expected since the imputation by Tangram is essentially aggregated from the single-cell gene expression showing high correspondence to spatial gene expression, and the single-cell transcriptomics might not agree with the spatial transcriptomics very well.

In summary, instead of making comparisons that are likely inconclusive, we believe it is better to conceive another study for developing a variation of GNTD to incorporate staining images or single-cell transcriptomics for imputation and a comprehensive evaluation of the performance in comparison with these methods in the future.

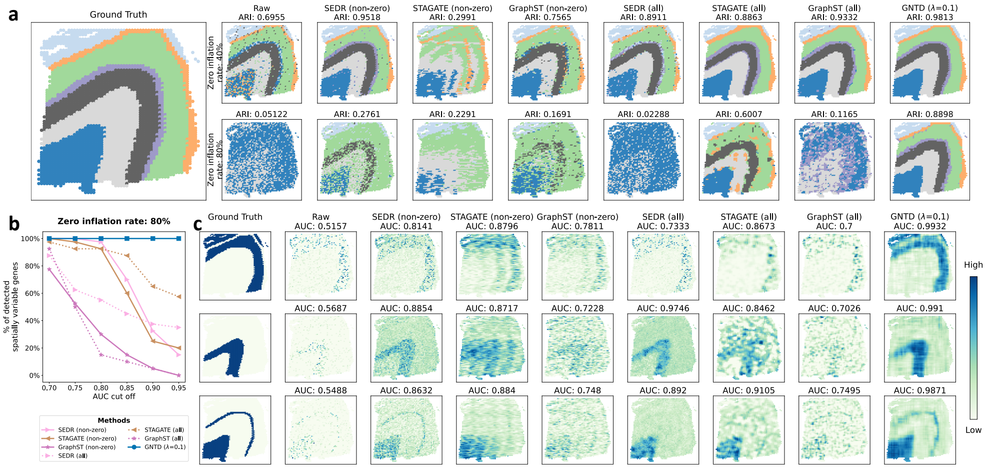

Fig. S1 Spatial domain detection and gene spatial pattern recovery by deep learning based models on simulated spatial transcriptomics data. a *Left:* Ground-truth segmentation of 6 cortical layers and white matter (WM) for simulated spatial transcriptomics data based on the annotation of the human dorsolateral prefrontal cortex (DLPFC) section 151673. *Right:* Visualization of the spatial domains detected by spot clustering on the raw and imputed spatial transcriptomics data (top: 40% zero inflation rates; bottom: 80% zero inflation rates), where the imputed data were obtained from SEDR, STAGATE, and GraphST with the best hyper-parameters in both non-zero only and all-entries training settings. **b** The percentage of correctly detected spatially variable genes by varying AUC thresholds on the simulated spatial transcriptomics data with 80% zero inflation rate. **c** Spatial pattern visualization of three example genes by their expression in the ground-truth, raw, and imputed spatial transcriptomics data for the simulation (80% zero inflation rate). Source data for **a** are provided as a Source Data file.

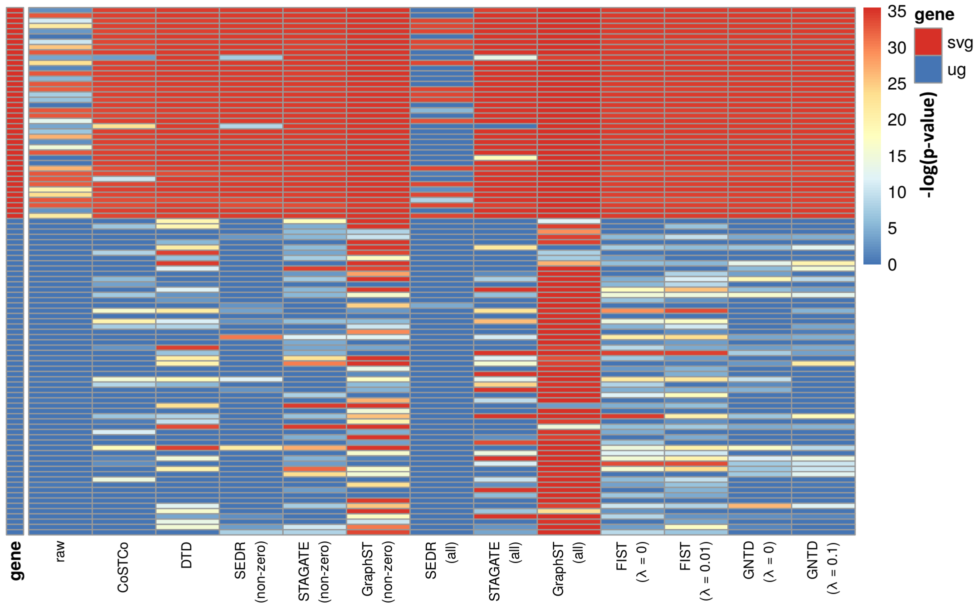

Fig. S2 Spatial variable genes detected from raw and imputated data by different models on simulated spatial transcriptomics data. Heatmap of spatial variation significance for both spatially variable (Red: svg) and ubiquitously expressed genes (Blue: ug) on raw and imputed spatial transcriptomics data for simulation (80% zero inflation rate), where −log(p-value) in each cell of heatmap is measured by spatially variable gene detection model SPARK. The bar on the left side of heatmap indicates ground-truth for gene types. Source data are provided as a Source Data file.

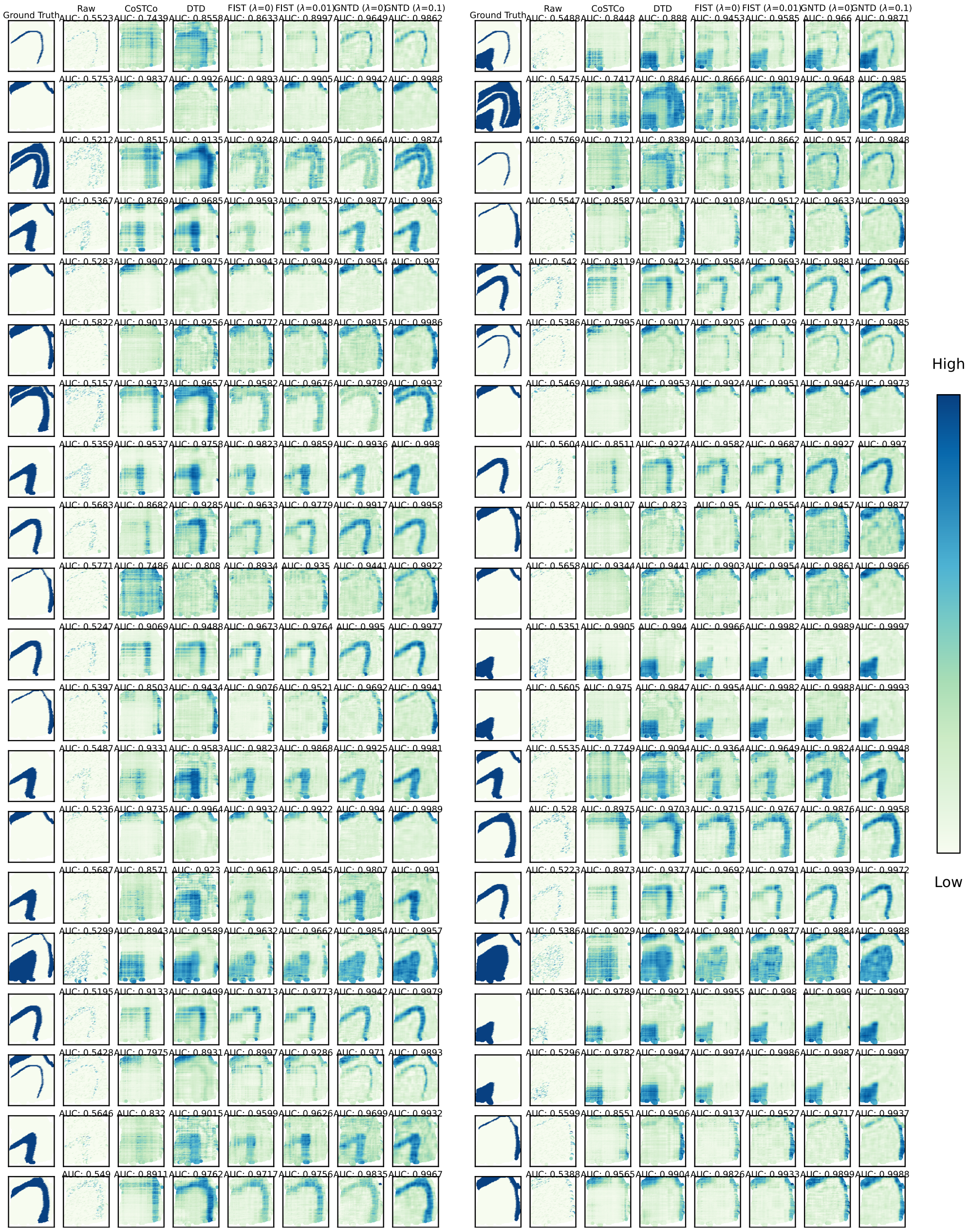

Fig. S3 Gene spatial pattern recovery for all spatially variable genes by tensor decomposition based models on simulated spatial transcriptomics data. Spatial pattern visualization of all spatially variable genes by their expression in the ground-truth, raw, and imputed spatial transcriptomics data for the simulation (80% zero inflation rate)

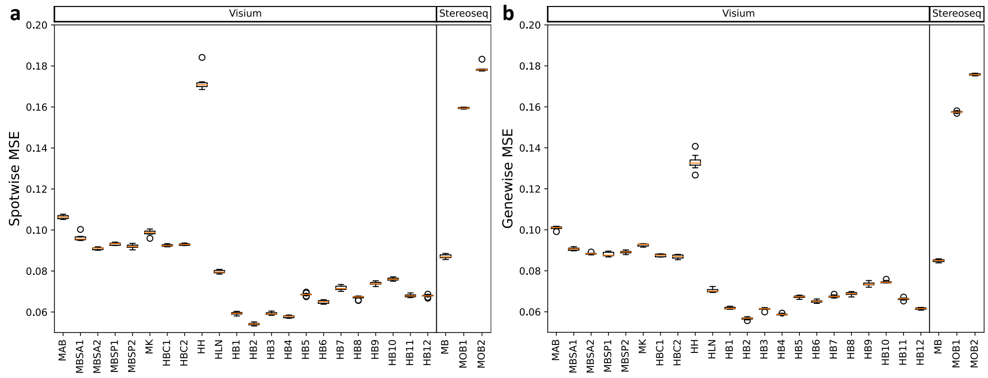

Fig. S4 Evaluation of imputation robustness on 22 Visium and 3 Stereo-seq datasets. **a** Box plot of spot-wise MSE of 10-fold cross-validation for GNTD imputation. (left: Visium; right: Stereo-seq) **b** Box plot of gene-wise MSE of 10 folds cross-validation for GNTD imputation. (left: Visium; right: Stereo-seq). In the boxplot in both **a** and **b**, the center line, box limits and whiskers denote the median, upper and lower quartiles and 1.5× interquartile range, respectively. Source data for **a** and **b** are provided as a Source Data file.

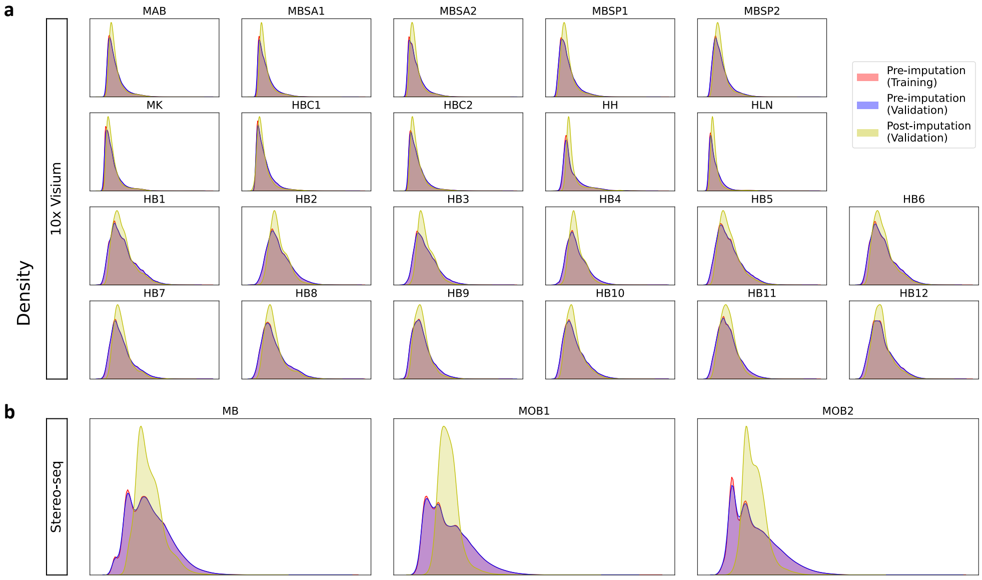

Fig. S5 Comparison between distribution of pre- and post-imputation on 22 Visium and 3 Stereo-seq datasets. The density distribution of expression values in the training set before (red) and after imputation (blue), and validation set after imputation (yellow) for **a** Visium and **b** Stereo-seq. Source data for **a** and **b** are provided as a Source Data file.

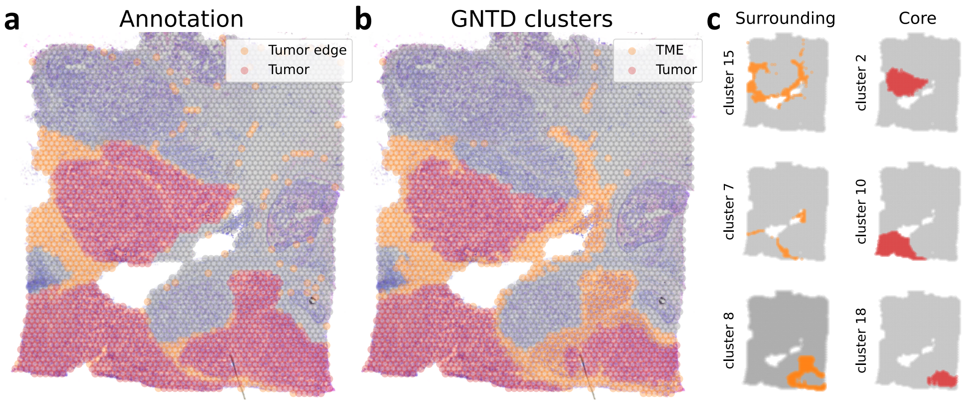

Fig. S6 Tumor-associated region segmentation on the human breast cancer tissue. a The annotation of 3 tumor regions (red) and their edges (yellow). **b** The corresponding regions detected in the spot clustering on GNTD imputation, and recognized as tumor (red) and tumor micro-environment (yellow). **c** The visualization of individual core and surrounding regions, and the cluster IDs on the left are matched with those in the supplementary table S2 for enrichment analysis on the human breast cancer tissue dataset.

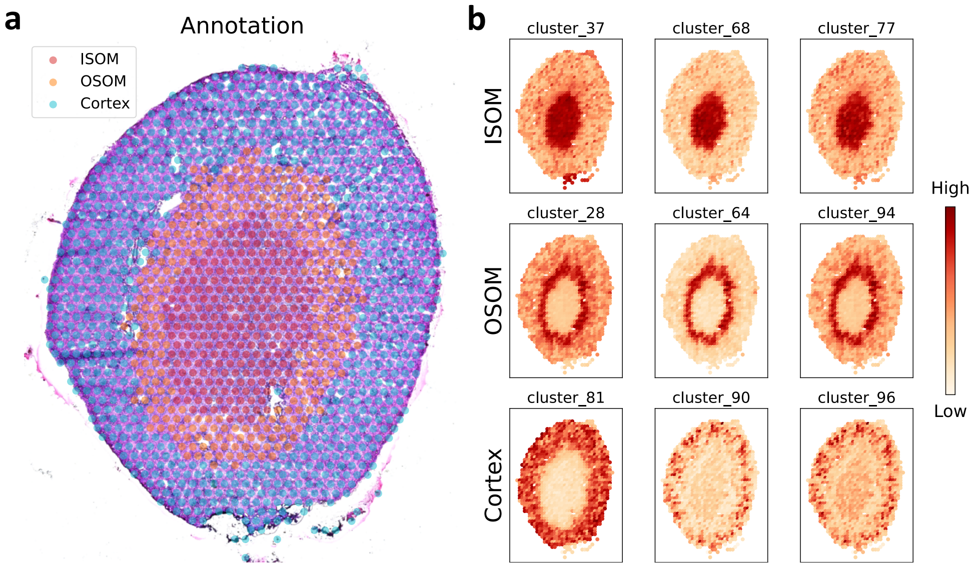

Fig. S7 Spatially co-expressed gene cluster detection on the mouse kidney tissue. a The annotation of 3 anatomical regions in the mouse kidney, including ISOM (red), OSOM (yellow), and Cortex (blue). **b** The visualization of spatially co-expressed patterns of selected gene clusters discovered by gene clustering on GNTD imputation, and gene clusters on each row highlight distinct regions in the mouse kidney tissue, and the cluster IDs on the top are matched with those in the supplementary table S3 for enrichment analysis on the mouse kidney.

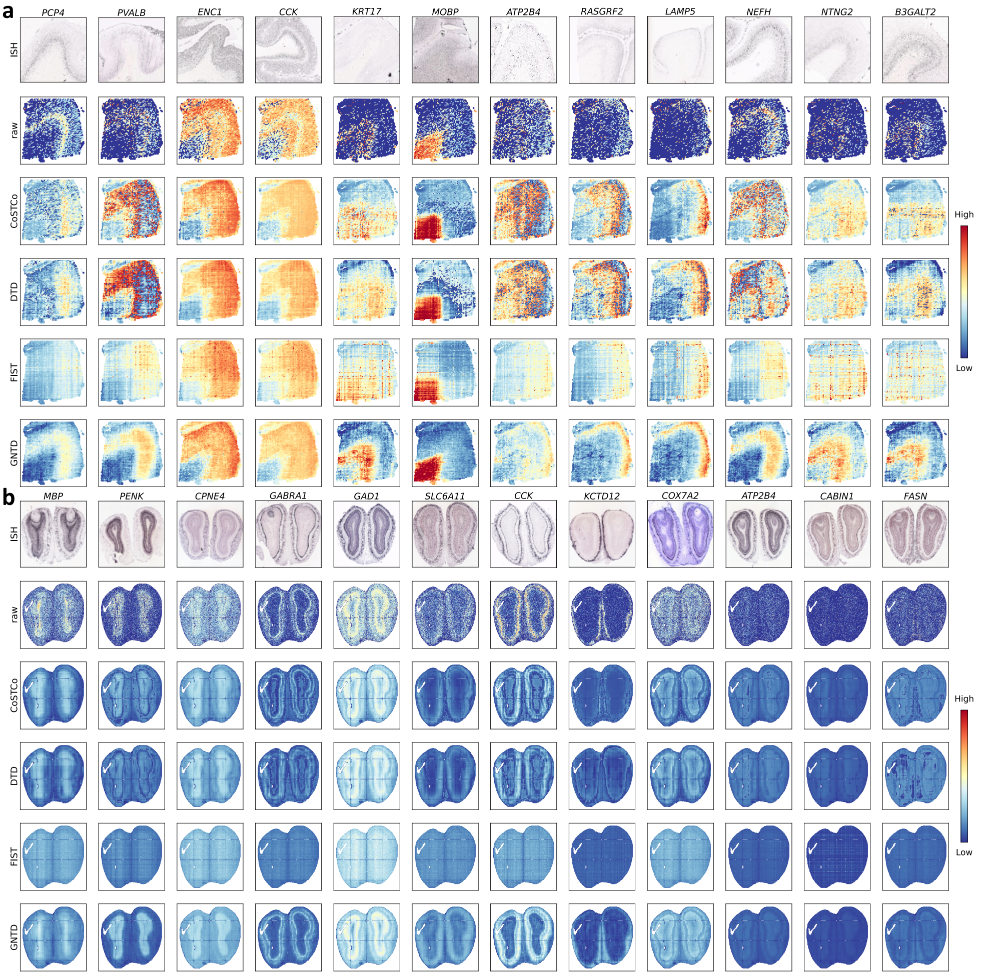

Fig. S8 Imputation for recovering the spatial patterns of marker genes on both Visium and Stereo-seq data (without color scaling). a The visualizations of 12 layer-specific marker genes (*PCP4*, *PVALB*, *ENC1*, *CCK*, *KRT17*, *MOBP*, *ATP2B4*, *RASGRF2*, *LAMP5*, *NEFH*, *NTNG2*, and *B3GALT*) of ISH images, raw data, and imputed data generated by four tensor-based models CoSTCo, DTD, FIST, and GNTD at their best rank on the DLPFC 151673 section from Visium. **b** The visualizations of 12 region-specific marker genes (*MBP*, *PENK*, *CPNE4*, *GABRA1*, *GAD1*, *SLC6A11*, *CCK*, *KCTD12*, *COX7A2*, *ATP2B4*, *CABIN1*, and *FASN*) of ISH images, raw data, and imputed data generated by four tensor-based models CoSTCo, DTD, FIST, and GNTD at their best rank on the mouse olfactory bulb section from Stereo-seq.

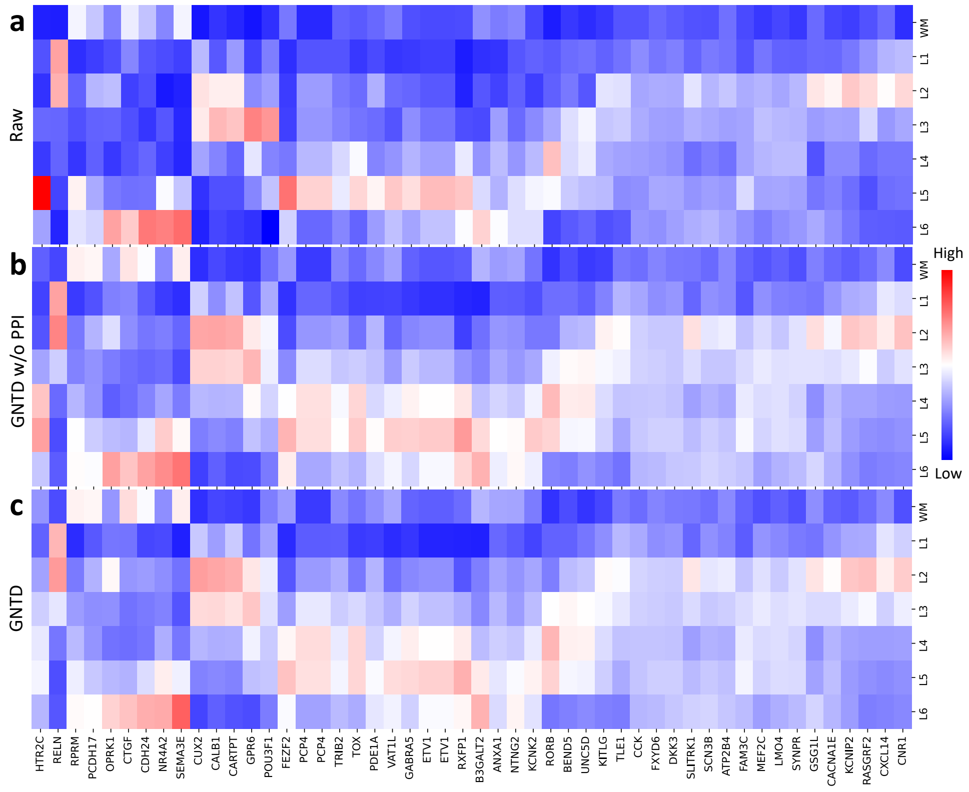

Fig. S9 Spatial expression patterns of marker genes for raw and imputation data by GNTD and its variant on the dorsolateral prefrontal cortex of the human brain. Heatmap of expression summary for 50 marker genes with known spatial patterns across 7 annotated layers for **a** raw, **b** GNTD without using PPI in the graph regularization (GNTD w/o PPI), and **c** GNTD on the DFLPC 151673 section, where each row represents either a specific cortical layer (L1-L6) or white matter (WM), and each column represents an individual marker gene. The value in each cell of the heatmap denotes the average expression of spots within the corresponding layer, and the color indicates the expression level. Source data for **a**, **b**, and **c** are provided as a Source Data file.

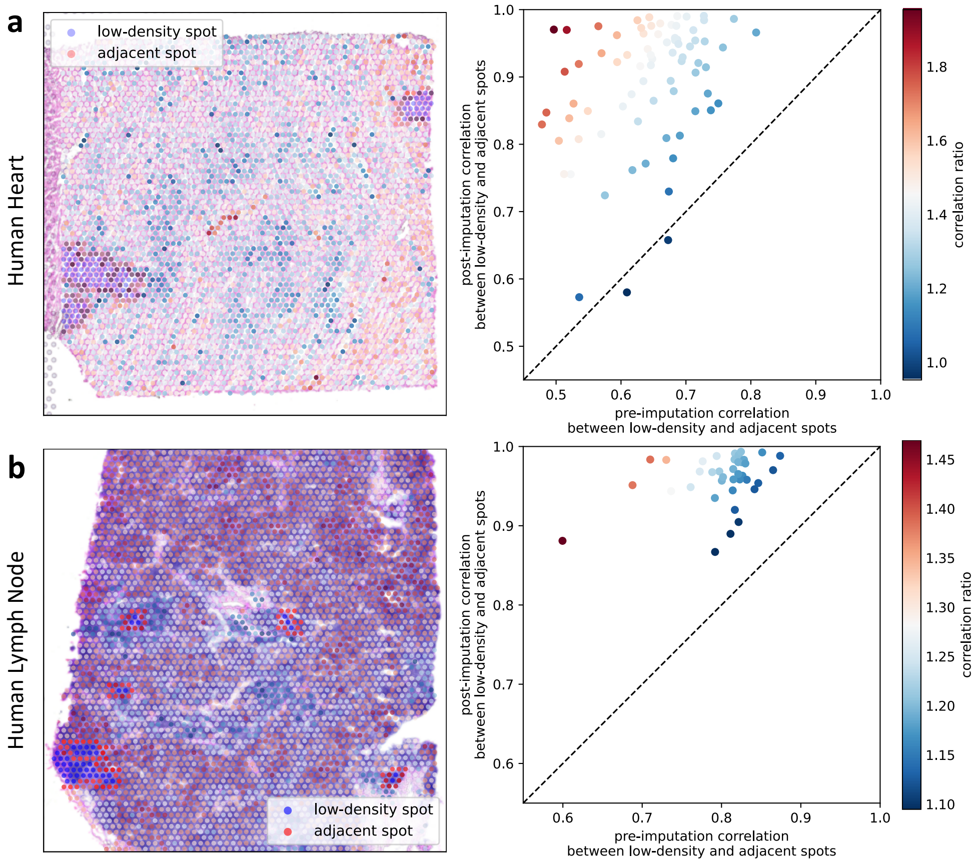

Fig. S10 Analysis of tissue regions with highly sparse expression in the Visium datasets. **a** Human heart and **b** human lymph node tissue sections were used in the analysis. For both tissues, *left*: The visualization of overall expression levels in the human heart based on the raw data, where spots in the low-density regions are labeled as red while their adjacent spots in the high-density regions are labeled as blue. *right*: The scatter plot of Pearson correlation coefficient between low-density spots and their high-density neighbors before (x-axis) and after imputation (y-axis) by GNTD, where each dot denotes one spot in the low-density regions and the corresponding color indicates the ratios between post- and pre-imputation Pearson correlation coefficients. Source data for **a** *right* and **b** *right* are provided as a Source Data file.

Table S1 Data summary

Project	Tissue	Species	Platform	# of spots	# of genes	Sparsity	Link
**10x Demo**	Mouse Adult Brain Section (MAB)	Mus Musculus	10x Visium	2698	11004	0.3934	Download
	Mouse Brain Sagittal-Anterior Section 1 (MBSA1)	Mus Musculus	10x Visium	2696	11004	0.3917	Download
	Mouse Brain Sagittal-Anterior Section 2 (MBSA2)	Mus Musculus	10x Visium	2825	11004	0.3481	Download
	Mouse Brain Sagittal-Posterior Section 1 (MBSP1)	Mus Musculus	10x Visium	3353	11004	0.3161	Download
	Mouse Brain Sagittal-Posterior Section 2 (MBSP2)	Mus Musculus	10x Visium	3293	11004	0.3060	Download
	Mouse Kidney Section (MK)	Mus Musculus	10x Visium	1438	11004	0.3781	Download
	Human Breast Cancer Block A Section 1 (HBC1)	Homo Sapiens	10x Visium	3813	17891	0.2648	Download
	Human Breast Cancer Block A Section 2 (HBC2)	Homo Sapiens	10x Visium	4015	17891	0.2615	Download
	Human Heart Section (HH)	Homo Sapiens	10x Visium	4235	17891	0.0964	Download
	Human Lymph Node Section (HLN)	Homo Sapiens	10x Visium	4039	17891	0.2741	Download
**spatialLIBD**	Human Brain DLPFC Section 151507 (HB1)	Homo Sapiens	10x Visium	4226	17891	0.0737	Download
	Human Brain DLPFC Section 151508 (HB2)	Homo Sapiens	10x Visium	4384	17891	0.0622	Download
	Human Brain DLPFC Section 151509 (HB3)	Homo Sapiens	10x Visium	4789	17891	0.0750	Download
	Human Brain DLPFC Section 151510 (HB4)	Homo Sapiens	10x Visium	4634	17891	0.0715	Download
	Human Brain DLPFC Section 151669 (HB5)	Homo Sapiens	10x Visium	3661	17891	0.0942	Download
	Human Brain DLPFC Section 151670 (HB6)	Homo Sapiens	10x Visium	3489	17891	0.0875	Download
	Human Brain DLPFC Section 151671 (HB7)	Homo Sapiens	10x Visium	4110	17891	0.0972	Download
	Human Brain DLPFC Section 151672 (HB8)	Homo Sapiens	10x Visium	4015	17891	0.0920	Download
	Human Brain DLPFC Section 151673 (HB9)	Homo Sapiens	10x Visium	3639	17891	0.1155	Download
	Human Brain DLPFC Section 151674 (HB10)	Homo Sapiens	10x Visium	3673	17891	0.1414	Download
	Human Brain DLPFC Section 151675 (HB11)	Homo Sapiens	10x Visium	3592	17891	0.0947	Download
	Human Brain DLPFC Section 151676 (HB12)	Homo Sapiens	10x Visium	3460	17891	0.1015	Download
**MOSTA**	Mouse Brain Section (MB)	Mus Musculus	Stereo-Seq	34654	10891	0.1018	Download
	Mouse Olfactory Bulb Section 1 (MOB1)	Mus Musculus	Stereo-Seq	27502	10891	0.1247	Download
	Mouse Olfactory Bulb Section 2 (MOB2)	Mus Musculus	Stereo-seq	27025	10891	0.1240	Download

Table S2 Biological processes enriched by differentially expressed genes for core and surrounding regions in human breast cancer section (p-values calculated from one-sided hypergeometric test)

**Region**	**Biological Process** (P-value)
**Core**	R-HSA-1280215 - Cytokine Signaling In Immune System (cluster 2: 5.38e-4; cluster 18: 6.03e-3; cluster 10: 2.21e-3)
	R-HSA-6785807 - Interleukin-4 And Interleukin-13 Signaling (cluster 10: 5.53e-3)
	R-HSA-9020702 - Interleukin-1 Signaling (cluster 10: 2.87e-2)
	R-HSA-913531 - Interferon Signaling (cluster 18: 6.62e-3; cluster 2: 8.75e-3)
	R-HSA-909733 - Interferon Alpha/Beta Signaling (cluster 18: 1.02e-5; cluster 2: 9.67e-3)
	GO:0019221 - cytokine-mediated signaling pathway (cluster 10: 2.65e-6; cluster 2: 1.16e-5)
	GO:0006090 - pyruvate metabolic process (cluster 10: 1.75e-5; cluster 18: 3.68e-4; cluster 2: 4.28e-4)
	GO:0009060 - aerobic respiration (cluster 10: 3.64e-3; cluster 2: 2.41e-2; cluster 18: 3.92e-2)
	GO:0033209 - tumor necrosis factor-mediated signaling pathway (cluster 10: 8.12e-3; cluster 18: 9.82e-3;; cluster 2: 4.57e-2)
	GO:0060337 - type I interferon signaling pathway (cluster 2: 2.05e-5; cluster 2: 6.78e-3)
	GO:0038061 - NIK/NF-kappaB signaling (cluster 10: 2.52e-2)
	KEGG - Neutrophil extracellular trap formation (cluster 2: 6.51e-3)
	KEGG - Necroptosis (cluster 10: 1.48e-4; cluster 18: 2.05e-3; cluster 2: 1.05e-2)
	KEGG - JAK-STAT signaling pathway (cluster 10: 3.57e-2; cluster 2: 4.01e-2)
	WP2884 - NRF2 pathway (cluster 10: 7.29e-5; cluster 18: 9.07e-4; cluster 2: 1.56e-3)
	WP111 - Electron Transport Chain (cluster 10: 4.22e-7; cluster 2: 3.06e-6; cluster 18: 2.17e-5)
**Surrounding**	R-HSA-6785807 - Interleukin-4 And Interleukin-13 Signaling (cluster 8: 2.93e-15; cluster 15: 3.19e-14; cluster 15: 1.41e-12)
	R-HSA-3000178 - ECM Proteoglycans (cluster 7: 6.73e-13; cluster 8: 4.13e-12; cluster 15: 9.6e-12)
	R-HSA-6783783 - Interleukin-10 Signaling (cluster 8: 7.59e-7; cluster 7: 2.01e-6; cluster 15: 2.95e-6)
	R-HSA-389948 - PD-1 Signaling (cluster 8: 3.75e-5; cluster 7: 1.66e-04; cluster 15: 2.07e-4)
	R-HSA-194315 - Signaling By Rho GTPases (cluster 8: 3.10e-5; cluster 15: 4.27e-4; cluster 7: 8.78e-4)
	GO:0030198 - extracellular matrix organization (cluster 8: 1.15e-49; cluster 15: 1.81e-43; cluster 7: 3.06e-43)
	GO:0030199 - collagen fibril organization (cluster 8: 1.02e-23; cluster 7: 1.59e-19; cluster 15: 4.59e-19)
	GO:0070098 - chemokine-mediated signaling pathway (cluster 8: 1.12e-13; cluster 7: 9.18e-12; cluster 15: 1.73e-11)
	GO:0048247 - lymphocyte chemotaxis (cluster 8: 2.56e-10; cluster 7: 1.02e-9; cluster 15: 1.69e-9)
	GO:0010818 - T cell chemotaxis (cluster 8: 5.91e-4; cluster 7: 1.51e-3; cluster 15: 1.72e-2)
	GO:0048246 - macrophage chemotaxis (cluster 8: 1.07e-3; cluster 7: 2.67e-3; cluster 15: 3.05e-3)
	KEGG - IL-17 signaling pathway (cluster 15: 1.22e-8; cluster 7: 8.82e-8; cluster 8: 2.95e-7)
	KEGG - HIF-1 signaling pathway (cluster 15: 6.95e-6; cluster 7: 1.14e-5; cluster 8: 2.37e-5)
	KEGG - RAP1 signaling pathway (cluster 15: 7.18e-6; cluster 18: 8.19e-6;; cluster 7: 1.64e-5)
	KEGG - Regulation of actin cytoskeleton (cluster 8: 2.67e-3; cluster 15: 5.92e-3; cluster 7: 6.29e-03)
	WP2806 - Complement system (cluster 7: 2.56e-10; cluster 15: 2.16e-9; cluster 8: 2.98e-9)
	WP3942 - PPAR signaling pathway (cluster 8: 5.98e-7 cluster 15: 5.11e-6; cluster 7: 1.18e-5)

Table S3 Biological processes enriched by genes with similar spatial patterns for different regions in mouse kidney section (p-values calculated from one-sided hypergeometric test)

**Region**	**Cluster**	**Biological Process** (P-value)
**ISOM**	**Cluster 37**	GO:0042632 - cholesterol homeostasis (4.53e-04)<br>GO:0055092 - sterol homeostasis (4.64e-04)<br>GO:0055088 - lipid homeostasis (1.22e-03)
	**Cluster 68**	GO:0006090 - pyruvate metabolic process (1.26e-04)<br>GO:0006757 - ATP generation from ADP (2.51e-03)<br>GO:0042866 - pyruvate biosynthetic process (2.69e-03)<br>GO:0046031 - ADP metabolic process, (2.94e-03)<br>GO:0006754 - ATP biosynthetic process (5.76e-03)
	**Cluster 77**	GO:0006163 - purine nucleotide metabolic process (4.19e-03)<br>GO:0019693 - ribose phosphate metabolic process (4.28e-03)<br>GO:0072521 - purine-containing compound metabolic process (6.74e-03)<br>GO:0009150 - purine ribonucleotide metabolic process (1.45e-02)<br>GO:0009259 - ribonucleotide metabolic process (1.60e-02)
**OSOM**	**Cluster 28**	GO:0006631 - fatty acid metabolic process (1.89e-04)<br>GO:0006633 - fatty acid biosynthetic process (5.13e-03)<br>GO:0016054 - organic acid catabolic process (1.25e-02)<br>GO:0046395 - carboxylic acid catabolic process (1.25e-02)<br>GO:0044282 - small molecule catabolic process (2.55e-02)
	**Cluster 64**	GO:0046942 - carboxylic acid transport (1.39e-04)<br>GO:0015849 - organic acid transport (1.40e-04)<br>GO:0015718 - monocarboxylic acid transport (3.21e-04)<br>GO:0015711 - organic anion transport (4.58e-04)
	**Cluster 94**	GO:1901605 - alpha-amino acid metabolic process (1.32e-05)<br>GO:0006520 - cellular amino acid metabolic process (7.16e-05)<br>GO:0006732 - coenzyme metabolic process (2.18e-04)<br>GO:0003091 - renal water homeostasis (3.14e-02)<br>GO:0003014 - renal system process (3.35e-02)<br>GO:0061318 - renal filtration cell differentiation (3.40e-02)<br>GO:0070293 - renal absorption (3.91e-02)
**Cortex**	**Cluster 81**	GO:1901605 - alpha-amino acid metabolic process (8.81e-07)<br>GO:0006520 - cellular amino acid metabolic process (2.93e-06)<br>GO:1901606 - alpha-amino acid catabolic process (4.79e-04)<br>GO:0009063 - cellular amino acid catabolic process (6.65e-04)<br>GO:0006790 - sulfur compound metabolic process (5.71e-03)
	**Cluster 90**	GO:0006939 - smooth muscle contraction (2.65e-05)<br>GO:0006940 - regulation of smooth muscle contraction (1.56e-04)<br>GO:1903524 - positive regulation of blood circulation (3.62e-04)<br>GO:1903522 - regulation of blood circulation (4.94e-04)<br>GO:0097746 - regulation of blood vessel diameter (1.98e-03)<br>GO:0050880 - regulation of blood vessel size (2.43e-03)<br>GO:0045907 - positive regulation of vasoconstriction (2.36e-03)<br>GO:0008015 - blood circulation (6.40e-03)
	**Cluster 96**	GO:0002028 - regulation of sodium ion transport (1.42e-03)<br>GO:0035725 - sodium ion transmembrane transport (1.96e-03)<br>GO:0015672 - monovalent inorganic cation transport (2.59e-03)<br>GO:0055067 - monovalent inorganic cation homeostasis (3.73e-03)<br>GO:0071805 - potassium ion transmembrane transport (6.15e-03)
